# POORER DIET QUALITY OBSERVED IN OLDER ADULTS WITH A GREATER NUMBER OF CHRONIC DISEASES

**DOI:** 10.1093/geroni/igz038.974

**Published:** 2019-11-08

**Authors:** Jessica L Krok-Schoen, Stephanie Fanelli, Janell Pisegna, Owen J Kelly, Christopher A Taylor

**Affiliations:** 1 School of Health and Rehabilitation Sciences, The Ohio State University, Columbus, Ohio, United States; 2 Abbott Nutrition, Columbus, Ohio, United States

## Abstract

Unhealthy lifestyle behaviors, including poor diet over many years, contribute to the development of chronic diseases, especially overweight/obesity, hyperglycemia, hypercholesterolemia and hypertension. Because poor diet is common to the diseases, it supports the notion of concurrently managing comorbidities through improved diet. Therefore, the purpose of this study was to assess differences in diet quality and nutrient intakes, in adults aged 65 years and older, by the number of chronic conditions. Data from 7,169 adults, aged 65 years and older, from the 2005-2016 National Health and Nutrition Examination Survey were assessed for selected chronic diseases from laboratory data: overweight/obesity (body mass index >25); hyperglycemia (glycated hemoglobin >5.7%); hypercholesterolemia (total cholesterol >200 mg/dL); hypertension (blood pressure >120/80 mmHg). The number of chronic diseases was computed per participant. Dietary intakes and diet quality score were assessed using 24-hour dietary recalls. Few adults had none of the selected chronic disease (n=79;1.4%), with others presenting 1 indicator (n=677;9.8%), 2 indicators (n=1,762;25%), 3 indicators (n=2,741;38.9%) and all 4 indicators (n=1,910;24.9%). Diet quality was significantly lower in those with three or four chronic diseases (P<0.001). Adults without any of the selected chronic diseases consumed significantly more calories, carbohydrates, fiber and added sugars, as well as folate, vitamin C and calcium than those with chronic diseases (P<0.001). Overall, dietary intakes from the day of intake were different for those with or without chronic diseases. These findings strengthen the need to promote healthy eating in older adults with one or more chronic conditions to help improve outcomes.

